# Pathological Anatomy

**Published:** 1836-07-01

**Authors:** 


### PATHOLOGICAL ANATOMY.

Illustrations of the Elementary Forms of Disease. By *Robert Carswell*, M.D. Professor of Pathological Anatomy in the University of London, &c. &c. Fasciculus Tenth. Atrophy.

The subject of this Fasciculus is an important one, and a connected view of the different modes and sources of atrophy, in the different organs and tissues of the body, cannot fail to interest as well as to instruct.

The definition of atrophy is simple, though not strictly correct:—it is the diminution in the quantity of the solid materials of the part, below the normal standard. Decrease of bulk constitutes, therefore, the essential physical character of atrophy, which is occasioned by a defective exercise of the function of nutrition.

Dr. Carswell glances first at the nature, origin, and causes of atrophy in general; and then treats, seriatim, of atrophy arising from a diminished supply of blood—atrophy from the diminished exercise of the function of innervation—and, finally, atrophy from the diminished exercise of the functions of an organ.

### Nature, Origin, and Causes of Atrophy.

Atrophy occurs under a great variety of circumstances. What are called monstrosities are often only forms of congenital atrophy, the development of particular organs or parts being at some time arrested in the embryo. Atrophy is in some instances a natural process. The organs required for the fœtal mode of existence, which are not essential after birth, waste, and are partially or totally absorbed. Thus the ductus arteriosus and the umbilical vein become converted into impervious cords. The vesicula alba and the omphalo-mesenteric vessels, which exist in the earlier periods of the embryo, are lost before birth. The thymus gland diminishes after birth and finally almost disappears. All these are examples of natural atrophy.

There is another kind of atrophy, the result of the laws imposed by nature, independently of actual morbid processes. It is the wasting of organs or of component parts of them, which occurs in advanced life, and makes the gradual decay of the organic being. An important instance of this kind of atrophy is cited by Dr. Carswell. It is well known, he says, that the solid bulk or weight of the lungs in old people suffers very great diminution, in consequence of atrophy of the walls of the air-cells and minute bronchial tubes, a change which may take place in separate portions or throughout the greater part of both lungs, and which may be carried to such an extent as to efface almost entirely the vesicular structure of these organs. The tendency to this kind of atrophy appears to manifest itself soon after the adult period of life, by an increase in the dimensions of the air-cells, which have become considerably larger than they are in youth, and much more so than in childhood, the increase in their capacity being, however, accompanied by a corresponding diminution in the thickness of their walls. As the atrophy advances with the increase of age, portions, or the whole of the walls of a greater or less number of the continuous air-cells and corresponding bronchial tubes disappear, and give rise to the formation of a loose, irregular, cellular structure, in which no trace of the vesicular character of the lungs is perceptible. The cells thus formed vary from the size of hemp-seed to that of a pea or more, and are intercepted by imperfect septa, on which but few bloodvessels are conspicuous. The substance of the lungs is of a dark colour, from the languid circulation of the blood through them, and the imperfect decarbonization of this fluid, as a necessary consequence of these changes, and which also extend their influence to the form and dimensions of the thorax, both of which, as well as the functions of respiration and circulation, and the temperature of the body, are variously modified.

It is questionable, perhaps, whether atrophy is a term fairly applicable to this dilatation of the pulmonary cells—a dilatation clearly the consequence of mechanical agency and of weakened vital powers. Provided, however, the student understands the nature of the alteration, and does not allow words to influence his ideas improperly, it matters not if the affection be denominated atrophy or not.

Dr. Carswell proceeds to state that, according to M. Ribes, the spongy structure of the penis undergoes changes, analogous to those observed in the lungs. The spleen is often much diminished in size, when it is hard, and composed chiefly of a cellulo-fibrous tissue and obliterated blood-vessels. Glandular organs, more especially the testes, lymphatic and mammary glands, are greatly reduced in size in some old people. The uterus is shrunk and very hard, and the ovaries are often transformed into a small mass of corrugated cellulo-fibrous tissue. The bones in general lose much of their weight, and become spongy and fragile; portions of some thick flat bones are reduced to a thin diaphanous plate; and fracture of the neck of the thigh-bone appears sometimes to have occurred in consequence of senile atrophy. The brain, spinal cord, and nerves participate in the general decay of old age; the brain, after seventy, according to Desmoulins, being diminished from one fiftieth to one-twentieth of its average weight, at the same time that it is specifically lighter than at forty years of age, and that the trunks and branches of the nerves are considerably less in size than in the adult. The longitudinal and transverse diameters of the brain in old age are stated by Cazauviehle to be several lines less than in the adult, and that the size of the corpora striata, optic thalami, and pons varolii, is sensibly diminished, whilst that of the cerebellum remains unaltered, even at the most advanced period of life. The muscles, especially those of voluntary motion, waste in old age, and the vascular system suffers in its turn and in its way.

Atrophy, occurring after birth, may either affect the whole body, when it is termed emaciation, marasmus, &c.—or it may take place in particular parts, as a permanent and bonâ fide pathological condition. The former is merely a symptom, the consequence of various affections—the latter it is which deserves methodical consideration.

Local atrophy, then, may, in reference to the causes which give rise to it, be divided into three forms:—1, atrophy from a diminished supply of blood; 2, atrophy, from the diminished exercise of the function of innervation; 3, atrophy, from the diminished exercise of the functions of an organ.

### I. ATROPHY FROM A DIMINISHED SUPPLY OF BLOOD.

It would be natural to anticipate à priori, that a diminution in the quantity of arterial blood required for the growth or perfect performance of the functions of an organ would be likely to occasion atrophy. This is found to be the fact. More or less atrophy of the brain occurs in some cases of ossification of the carotid and vertebral arteries within the cranium; in the spleen, the uterus, &c. from the same morbid condition of their respective arteries; and not unfrequently in the inferior extremities, from this and other diseased states which interrupt the circulation of the blood through the larger arteries of these parts. Complete atrophy of the testicles follows obliteration, from disease, of the spermatic arteries, and from ligature of these vessels for the cure of varicocele. Tumors are sometimes treated on this principle; their main arteries being tied, or attempts being made to effect more or less obliteration of their vessels by punctures, incisions, and so forth.

Compression, from interruption to the capillary circulation, occasions an atrophied condition of the part compressed. The feet in some women, especially the Chinese, the inferior part of the thorax in females who lace tightly, are instances of this description. In some old women, who secure their petticoats around their waists by a string, a deep groove is found on the surface of the liver, sometimes from half an inch to an inch in depth. The compression exercised by tumors is known well enough to occasion it. The ribs or the vertebræ may be extensively absorbed from the pressure of an aneurysm, or of a morbid growth. Serous cysts in the kidney, or accumulations of urine or of pus in its pelvis or infundibula, occasionally produce an almost total disappearance of the glandular structure.

Dr. Carswell describes the atrophied state of the brain, not very uncommon in old persons. But, in our notice of Dr. Sims's paper,* we alluded so fully to the subject, that we need not re-enter on its consideration at present. Atrophy of the spinal cord is observed in conjunction with atrophy of the cerebral convolutions, and depends on the same causes.

"The accumulation of air in the vesicular structure of the lungs, known under the appellation of pulmonary emphysema, gives rise on the same principle to a particular form of atrophy of these organs. Thickening of the mucous membrane of the smaller bronchi in particular, the retention of inspissated mucus, compression and stricture of these tubes, violent and frequently repeated attacks of coughing, and efforts of every kind which prevent the free exit of the inspired air, are the causes of this form of atrophy. Acting as mechanical obstacles, they impede the transmission of the air through the bronchi both during inspiration and expiration; but the muscles of the former being much more powerful in their operation than those of the latter, introduce a greater quantity of air into the air-cells than can be expelled at the subsequent expiration, which therefore gradually accumulates in them, distends them mechanically and ruptures them, or by the compression which it exercises on their capillary circulation, destroys them by a slow process of atrophy. It is only, however, when the emphysema is great in degree that atrophy contributes to its production, or rather its further increase. This is best seen in those cases in which a portion of emphysematous lung projects considerably, as frequently happens when it occupies the summit or anterior border of this organ. When emphysema depends simply on dilatation of the air-cells, it may occupy only a portion or the whole of one or both lungs; but when rupture of the air-cells has taken place to a great extent, whether from distention or atrophy, it is generally confined to a limited number of lobules. In the former case the air-cells acquire the size of millet-seed, hemp-seed, or a small pea, the bulk of one or both lungs being at the same time, proportionally increased; in the latter, several of them, or even a few contiguous lobes are thrown into one cavity, sometimes sufficiently large to contain a pigeon's egg, presenting a number of laminated and filamentous intersections, which divide it partially into irregular compartments. It is, therefore, as I have stated, only in this variety of pulmonary emphysema, which is a chronic affection, that atrophy is conspicuous. It is not observed in interlobular emphysema, which is an acute affection, as the air is absorbed before it has time to produce atrophy by compression." 6.

Dr. Carswell goes on to state, that there is a form of dilatation of the bronchi which presents a remarkable example of atrophy. It sometimes, he says, occupies bronchi of considerable size, but much more frequently the small ramifications or terminations of these, which are dilated into the form of globular sacs, varying from the size of a pea to that of a walnut. Some of them consist of a uniform smooth cavity; others present several anfractuosities, which generally happens when a number of contiguous tubes communicate with each other in consequence of complete atrophy of a portion of their walls. In this case they might be confounded with emphysema, were it not for the nature of their contents, which consist always of a viscid, straw-coloured, muco-puriform secretion. The tenuity of their walls is such, that Laennec has aptly compared them to the outer skin of an onion, and through which the colour and vesicular structure of the lung is seen as distinctly as beneath the transparent pleura. Their lining or mucous membrane is re-

---

* Vide No. 47.

markably smooth, sometimes of a red, but more generally of its natural colour, and is much more tenacious than healthy mucous tissue. This form of dilatation is generally confined to a small number of bronchial tubes: but it sometimes affects the greater part of a lobe, generally the upper, the vesicular structure of which is compressed, deprived of air, and in that state of flaccidity which it presents in the case of chronic pleurisy with effusion.

The last mode in which atrophy is occasioned, by obstruction to the circulation of blood in the capillaries—the last mode, at least, which requires particular notice, is a curious one. It is the formation of a contractile fibrous tissue on the surface of some organs, and in the interior of others. Thus, in chronic pleurisy, lymph and serous or purulent fluids are poured out, occupying the pleural cavity, and investing the surface of the pleura pulmonalis. The fluid may be absorbed, but the lymph on the pulmonary pleura remains. It becomes organized and ultimately converted into a strong fibrous membrane partially or generally covering the pleura, and producing partial or general compression, and atrophy of the pulmonary tissue.

In chronic peritonitis the same compression, and the same bad consequences follow the same cause. The stomach, intestines, and spleen seem girt by an unyielding cincture of fibrous tissue, binding them to the spine, limiting their capacity, and giving rise to the effects of stricture more or less complete on the intestinal canal. The spleen not unfrequently becomes individually surrounded with a thickened and fibrous capsule, when it is usually seen of small size. This capsule may be converted into cartilage or bone.

Dr. Carswell places in the same category stricture of the œsophagus and intestines, resulting from the cicatrization of ulcers that have affected their muscular tunic. He remarks that he has met with several fatal cases of stricture of the intestines produced by cicatrices formed of this tissue, and succeeding to ulceration, originally situated in the glands of Peyer, which had destroyed the muscular coat around the whole circumference of the tube in that portion of it occupied by these glands. The patients had left the hospital in a state of imperfect convalescence from typhoid fever, and returned some months, in one case more than twelve months after, with all the symptoms of ileus, the progressive development and increasing severity of which, as the constriction of the tube advanced, were strikingly illustrated by the history of the cases, and left no doubt as to the nature and cause of the disease. We may pause to observe that the term atrophy has a large grasp in modern pathology. Diminution of substances, no matter how occasioned, receives this name. The student would be at first surprised if he heard that a lung compressed by pleurisy, and apparently dilated by enlarged bronchial tubes, was equally considered atrophic—and when he passed from the dilated bronchus to a strictured œsophagus, and was informed that he witnessed atrophy still, his wonderment would probably increase. But a little consideration would shew him that in strict propriety, and in its wide acceptation, atrophy exists in all these cases, and he would perceive the advantage of these pathological generalizations, which serve to correct by enlarging our views of the effects of morbid actions. Yet this very circumstance renders the present work more adapted for the man who has made some advances in the science, than for the student who is just upon its threshold.

Our author instances as a remarkable example of the atrophy of organs from the development of the contractile fibrous tissue in their interior, that affection of the liver which has been designated cirrhosis. His description of its appearance and its causes is precise, but would be scarcely understood by those who are unacquainted with the terms employed by Mr. Kiernan in his account of the anatomy of the organ. We shall therefore give only the principal heads of Dr. Carswell's statements.

The liver, when affected with atrophy from this cause, is sometimes reduced to a fourth of its normal dimensions; its consistence generally increases with the diminution of its bulk; it appears shrunk, and has an irregularly rounded form, particularly at its edges, and the whole of its external surface is raised into round flat projections, varying from the size of hemp-seed to that of a pea, or even a small cherry. Examined more narrowly, the round flat projections are found to be composed of several smaller ones, and these, again, of the individual lobules of the liver; so that the larger projections are formed of aggregated groups of lobules, each separated the one from the other by cellulo-fibrous or fibrous tissue, the quantity of which varies considerably, and is always greatest between the largest groups of lobules. On making a section of the liver, the fibrous tissue is found to form a conspicuous feature in it: for its quantity is greater than that of the lobular structure, and its white or grey colonr contrasts strongly with the rust, yellowish, or greenish brown tint of the lobules. On pursuing the examination with more accuracy and minuteness, it is found that the fibrous tissue surrounds the portal veins, occupying their sheath, and both compressing those veins themselves and the lobules of the organ into which they pass. In the earlier stage of the disease the quantity of fibrous tissue is, of course, comparatively small—it increases with the progress of the malady—and in some instances it ends by effecting the almost entire removal of the natural structure of the organ. Such is the sum of Dr. Carswell's laborious account of this disease, which, from the rust colour of the liver, was named by Laennec cirrhosis, and which he considered as depending on the development of a new tissue of a peculiar description. The different colours which the liver presents are derived from the varying quantities of blood and bile accumulated in the lobules. The rust-brown and orange colours are most common at the commencement, the greenish brown towards the conclusion.

"In some cases of cirrhosis, the obstruction to the circulation of the blood through the portal veins gives rise to appearances very different from those which I have described as characterising this affection. As these vessels are compressed and narrowed in the manner I have stated, they are generally found to contain only a small quantity of blood after death. It occasionally happens, however, that they are filled or distended with a mixture of coagulated blood, fibrine, and bile, the presence of the two former in particular indicating that the circulation must have almost entirely ceased befoie death. In three cases of this kind which I have met with, the whole of the portal system, from the commencement of its trunk to its termination in the lobules, was in this state. A great many of the portal veins, both large and small, were enormously dilated, and some of the smaller ones appeared to be ruptured, so that, when cut across, they presented the appearance of bloody tumours or fungous hæmatodes, scattered throughout the substance of the liver." 12.

The nature of cirrhosis affords a satisfactory explanation of the ascites, which, in greater or less quantity, is its constant attendant, and increases as it proceeds.

We now terminate the consideration of the first variety of atrophy, and proceed to that of the second.

### II. ATROPHY FROM THE DIMINISHED EXERCISE OF THE FUNCTION OF INNERVATION.

We do not know the kind nor the amount of influence exerted by the nervous mass on the function of nutrition. But that that influence is considerable, is proved by the atrophy that follows various diseases or injuries of the brain, spinal chord, or nerves. The wasting of the limbs or even entire side of the body in hemiplegia is well known, and greater than the mere disuse of the muscles on that side would account for. Compression of the main nerves of a limb produces extreme atrophy. This is seen after dislocations of the shoulder, in which the brachial plexus has been pressed on.

A remarkable case of atrophy of the right inferior extremity, from injury of the crural and sciatic nerves on that side, is recorded by Lobstein, which he observed in a man fifty-four years of age, who was thrown down in the street when a child. The right limb became soon after feeble, and gradually diminished in bulk as he advanced in years. The muscles, when examined after death, were pale, and reduced to the form of a fleshy membrane; the gastrocnemius and soleus muscles weighed only two ounces and six drachms, whilst those of the healthy limb weighed nearly eight ounces; the bones of the right side of the pelvis were considerably reduced in size and thickness; the right femur weighed only three ounces two drachms and a half, whilst that of the opposite side weighed nearly five ounces seven drachms.

### III. ATROPHY FROM THE DIMINISHED EXERCISE OF THE FUNCTIONS OF AN ORGAN.

The wasting of voluntary muscles when disused is too familiar to require to be dwelt on. The involuntary muscles waste by the same law under the same circumstances. In cases of artificial anus, the muscular tunic of that portion of intestine through which the aliment has long ceased to pass is reduced to a thin transparent membrane.

"Causes which permanently interrupt the passage of the air through the bronchi, as tumours which compress these tubes, accidental products contained within them, cicatrization and construction of their walls, or which prevent for a considerable length of time the free and full expansion of the lungs, as incidental fluid and solid products contained within the cavity of the pleura, vicious modes of dress, as well as those occupations and habits which limit the action of the respiratory muscles, or which retain these organs in a state of comparative inactivity, give rise to partial or general diminution of bulk of one or both lungs. When a portion of the bronchial tube becomes obliterated by any of the causes which have been enumerated, the remaining portion of it, or the terminal branches of which it is composed, frequently undergo a gradual diminution of bulk, become likewise obliterated, and are ultimately converted into solid fibrous cords. Sometimes only one bronchus, but more frequently several bronchi, either in one or both lungs, are found obliterated in this manner, and the portions of pulmonary tissue to which they are distributed entirely deprived of air, collapsed, flaccid, and atrophied. An interesting memoir has lately been published by my friend Mons. Reynaud, illustrating several of the forms of this kind of atrophy, or rather *obliteration* of the bronchi, which he has shewn to be much more frequent in its occurrence than had been supposed by other pathologists. The bronchial tubes thus effected are seldom larger than those of the third or fourth order, and, therefore, the atrophy which takes place in consequence of their obliteration is seldom accompanied by a marked diminution in the bulk of the lungs, unless several of them are simultaneously affected. When this happens, however, an obvious diminution of bulk of the affected lung is observable, the external surface of which presents an irregular, lobulated appearance, in consequence of the healthy portions of the pulmonary tissue into which the air is freely admitted, being raised above those from which it is excluded, or which are in a state of atrophy, from the impervious condition of their bronchi."

Not unfrequently, in children, the larger bronchial tubes are compressed by tuberculated glands. But although the respiratory function is interfered with, Dr. Carswell is not aware of atrophy of the pulmonary tissue having been observed. He saw, however, an instance of this in a monkey that he examined along with Mons. Reynaud. The left division of the trachea was surrounded by a mass of tuberculated bronchial glands as large as a walnut, in the centre of which it was imbedded, and so compressed as to be rendered completely impervious to the passage of the air. The corresponding lung was reduced to nearly one-fourth of the size of the other, and as the cavity of the pleura contained no fluid, the parietes of the chest on this side were depressed in the same manner, as in cases of chronic pleurisy with permanent compression of the lung, after absorption of the effused fluid. Both lungs were crowded with groups of tubercles of various sizes, and the right, that by means of which the function of respiration had been for some considerable time exclusively performed, was extensively emphysematous.

Atrophy of the gall-bladder from interference with the performance of its function has been often noticed. Dr. Carswell has seen it arise either from obliteration of the cystic duct by enlarged lymphatic glands, or from a preternatural communication existing between the gall-bladder and a neighbouring portion of intestine. In the first instance no bile got into the former; in the second it was prevented from accumulating in it.

" The remaining organs requiring notice, in which atrophy occurs in consequence of the suspension or cessation of their special functions, are those of generation, especially the mammæ and testes, the brain, spinal chord, and nerves. It is well known that the mammæ seldom acquire the same magnitude in females who have not suckled as in those who have; and the great diminution which they undergo, when the exercise of their function is no longer required, affords a striking illustration of the influence of the suspension of a physiological act, in the production of atrophy. The testes, also, furnish us with examples of atrophy equally remarkable, although not always equally well understood. In persons who have spent their youth under the humiliating and blighting influence of certain monastic vows, the testes either remain in a state of imperfect development or become wasted; and Baron Larrey asserts that injuries of the cerebellum, from wounds occupying the region of the occiput, are also followed, at some period after recovery, by atrophy of the testes. Although I am not aware that similar cases have been observed by other authors, it does not appear to me that there are any grounds for disbelieving the Baron's statement, more especially as it has been ascertained that the special function of these organs, if not dependent for its accomplishment on the physiological exercise of that of the cerebellum, is certainly either increased to a remarkable degree, or altogether controlled by various morbid conditions of this organ. Many examples have been recorded which prove that such a functional relationship exists between the testes and cerebellum; and I may add, in further confirmation of the fact, that I have met with two cases in particular, in which this relationship was manifested in a most remarkable manner. They occurred in two young men, from eighteen to twenty years of age, who reduced themselves to a state of the most appalling moral and physical degradation by the act of self-pollution. Both of them died from its effects; one of them having often declared that he was compelled towards the gratification of a desire which he had no power to control, for he had frequently attempted the consummation of it after the prepuce had been excised as a means of prevention, and when the glans and part of the penis were in a state of active inflammation. In each of these patients the cerebellum was the seat of a tumour as large as a hen's egg, composed entirely of the medullary sarcoma." 13.

These are certainly curious facts, and they appear to prove that the cerebellum is really concerned with the generative organs. Yet, on the other hand, abscesses and tumors have existed in the former without any material affection of the latter, and these may be disordered or diseased, without, apparently, disturbing the cerebellum. The most common cause of atrophy of the testes is masturbation. There can be no doubt of the frequency both of the vice and the consequence. We lately dissected two testes wasted from this cause. The proper fibrous tunic of the organ was rather thickened, as were its pillars. The lobules of the testis were diminished, but the tubuli were not, so far as we could see, diseased. In one testis (both were taken from the same individual) the globus minor of the epididymis was consolidated by thickening of the fibrous tissue, and the canal of the vas deferens was to all appearance impervious. On the whole we should say that the fibrous tissue both in the testis and epididymis was augmented, and that the glandular structure was greatly diminished. We could not succeed in injecting the rete in either testis; but those who know how frequently injections of the testicle are unsuccessful, will not attach much importance to this circumstance.

We have mentioned these particulars respecting the testis, because there are not many recorded dissections of the organ in a state of atrophy, Had we space we should feel disposed to relate a very interesting case, in which the testes, wasted to the dimensions of small nuts, were gradually restored to nearly their natural size and functions. That this fortunate result of treatment seldom happens, is due perhaps to the permanent contraction of the fibrous tissue which surrounds the tubular structure and permeates it.

Dr. Carswell, to whom we return, observes that there are no facts which prove that the brain, or particular portions of it, waste merely in consequence of diminished activity of function. It is only in cases of disease of the convolutions, that atrophy of the deeper parts is met with. In some of the instances of atrophy of the convolutions, which Dr. C. has seen occur in the general paralysis of the insane, the corpus striatum and optic thalamus were much diminished in size, either on one side or both, according as the former occupied one or both hemispheres ; nor was the secondary occurrence of atrophy confined to these portions of the brain ; it extended to the crura cerebri, pons Varolii, medulla oblongata, and spinal chord.

" I have met," says our author, " with two cases of a remarkable lesion of the spinal cord accompanied with atrophy. One of the patients was under the care of Mons. Louis, in the Hospital of La Pitié, the other under the care of Mons. Chomel, in the Hospital of La Charité, both of them affected with paralysis. I did not see either of the patients, but I could not ascertain that there was any thing in the character of the paralysis or the history of the cases, calculated to throw any light on the nature of the lesion found in the spinal cord. I have represented the appearances observed in one of these cases in Plate IV. *fig.* 4, in which the pons Varolii was also affected, and which convey an accurate idea of the physical characters of the lesion. In this case a distinct portion of the cord was affected with softening, which of itself would no doubt have accounted for the paralysis ; but in the other case there was no other lesion present than that to which I allude, to which the paralysis could be attributed. The anterior surface of the spinal cord presented a number of spots, from a quarter of an inch to half an inch in breadth, of an irregular form, of a yellowish brown colour, smooth, glossy, without vascularity or any alteration in the colour or consistence of the surrounding medullary substance. The medullary substance thus affected was very firm, somewhat transparent, and atrophied. At the root of the medulla oblongata, these changes occupied the whole breadth of both the medullary fasciculi to the extent of half an inch in breadth from above downwards. Further down, they were confined to distinct spots on each fasciculus, and several of the same kind, but smaller, occupied the pons Varolii. The depth to which the medullary substance was affected in this manner varied from half a line to three or four lines, and on dividing the cord, it was seen to penetrate as far as the grey substance." 14.

It is singular, that the nerves of muscular organs which have long been paralysed, seldom present any material diminution of their bulk. Atrophy has been more frequently observed in the optic than in any other nerve, in consequence of disease or lesion of the organ to which it is distributed. The extent of the atrophy varies with that of the lesion of the eye, as well as with its duration. The atrophy, in the great majority of cases, is confined to that portion of the nerve situated between the eye and the optic commissure; and in those cases in which it extends beyond the latter point, the most generally received opinion is, that it is the nerve on the opposite side that is affected. When the atrophied nerve is examined, it is found to be variously reduced in bulk ; the nervous matter in particular is very small in quantity, or has altogether disappeared. The cellular and fibrous tissues of the nerve, consequently, become more apparent, constitute, in some cases, the greater part of the nerve, or occupy, in others, its place, in the form of a fibro-cartilaginous cord.

This terminates the Fasciculus before us, at least it concludes the account of atrophy. The plates are, as usual, very beautiful, and highly creditable to the parties concerned. We may fairly say that the work has improved as it has proceeded—no mean recommendation. The author deserves all the encouragement that can possibly be bestowed upon him.

